# Cystathionine Beta-Synthase (CBS) Contributes to Advanced Ovarian Cancer Progression and Drug Resistance

**DOI:** 10.1371/journal.pone.0079167

**Published:** 2013-11-13

**Authors:** Sanjib Bhattacharyya, Sounik Saha, Karuna Giri, Ian R. Lanza, K. Sreekumar Nair, Nicholas B. Jennings, Cristian Rodriguez-Aguayo, Gabriel Lopez-Berestein, Eati Basal, Amy L. Weaver, Daniel W. Visscher, William Cliby, Anil K. Sood, Resham Bhattacharya, Priyabrata Mukherjee

**Affiliations:** 1 Department of Biochemistry and Molecular Biology, College of Medicine, Mayo Clinic Rochester, Minnesota, United States of America; 2 Department of Pathology, Stephenson Cancer Center, University of Oklahoma Health Science Center, Oklahoma City, Oklahoma, United States of America; 3 Division of Endocrinology, College of Medicine, Mayo Clinic Rochester, Minnesota, United States of America; 4 Department of Gynecologic Oncology, M. D. Anderson Cancer Center, Houston, Texas, United States of America; 5 Department of Cancer Biology, M. D. Anderson Cancer Center, Houston, Texas, United States of America; 6 Center for RNA Interference and Non-Coding RNA, M. D. Anderson Cancer Center, Houston, Texas, United States of America; 7 Department of Obstetrics and Gynecology, College of Medicine, Mayo Clinic Rochester, Minnesota, United States of America; 8 Department of Biostatistics and Bioinformatics, College of Medicine, Mayo Clinic Rochester, Minnesota, United States of America; 9 Department of Pathology, College of Medicine, Mayo Clinic Rochester, Minnesota, United States of America; 10 Department of Obstetrics and Gynecology, Stephenson Cancer Center, University of Oklahoma Health Science Center, Oklahoma City, Oklahoma, United States of America; Children's Hospital Boston & Harvard Medical School, United States of America

## Abstract

**Background:**

Epithelial ovarian cancer is the leading cause of gynecologic cancer deaths. Most patients respond initially to platinum-based chemotherapy after surgical debulking, however relapse is very common and ultimately platinum resistance emerges. Understanding the mechanism of tumor growth, metastasis and drug resistant relapse will profoundly impact the therapeutic management of ovarian cancer.

**Methods/Principal Findings:**

Using patient tissue microarray (TMA), *in vitro* and *in vivo* studies we report a role of of cystathionine-beta-synthase (CBS), a sulfur metabolism enzyme in ovarian carcinoma. We report here that the expression of cystathionine-beta-synthase (CBS), a sulfur metabolism enzyme, is common in primary serous ovarian carcinoma. The *in vitro* effects of CBS silencing can be reversed by exogenous supplementation with the GSH and H_2_S producing chemical Na_2_S. Silencing CBS in a cisplatin resistant orthotopic model *in vivo* by nanoliposomal delivery of CBS siRNA inhibits tumor growth, reduces nodule formation and sensitizes ovarian cancer cells to cisplatin. The effects were further corroborated by immunohistochemistry that demonstrates a reduction of H&E, Ki-67 and CD31 positive cells in si-RNA treated as compared to scrambled-RNA treated animals. Furthermore, CBS also regulates bioenergetics of ovarian cancer cells by regulating mitochondrial ROS production, oxygen consumption and ATP generation. This study reports an important role of CBS in promoting ovarian tumor growth and maintaining drug resistant phenotype by controlling cellular redox behavior and regulating mitochondrial bioenergetics.

**Conclusion:**

The present investigation highlights CBS as a potential therapeutic target in relapsed and platinum resistant ovarian cancer.

## Introduction

In recent years the gasotransmitter H_2_S has gained immense importance ranging from prokaryote to vertebrate biology and expanding [1]–[6]. In a seminal article, Roth et al. demonstrated that pre-treatment with H_2_S prevented hypoxic injury in mice by drastically reducing the animal’s core body temperature and metabolism, akin to what is observed in hibernating mammals [7]. Yet another article demonstrated that loss of H_2_S synthesizing enzymes sensitized a plethora of disease causing bacteria to antibiotics mainly through increased oxidative stress [8]. However, a role for metabolic enzymes that synthesize H_2_S has not been described in cancer biology remains under investigated.

In humans, two main metabolic enzymes synthesize H_2_S,cystathionine beta synthase (CBS) primarily localized in the brain and liver tissues and cystathionine gamma lyase (CSE/CTH) primarily found in muscle tissues [9]. CBS is the first rate-limiting enzyme in the transsulfuration pathway and by utilizing homocysteine (Hcy) produces H_2_S and the cysteine precursor cystathionine [10]. Besides cellular uptake of cystine, cysteine synthesis is the rate-limiting step for glutathione (GSH) production, the ubiquitous antioxidant. Studies using CBS knockdown mice have underscored the importance of this enzyme in cardiovascular and neurovascular disorders, primarily causing endothelial dysfunction, thought to be due to enhanced plasma Hcy levels [11]–[13]. However, supplementation with Vitamin B_12_ and folic acid (which facilitate remethylation of Hcy to methionine) reduced circulating Hcy levels yet failed to reduce the symptoms of cardiovascular disease. On the other hand, Vitamin B_6_, a cofactor for CBS, failed to reduce circulating Hcy levels in recent clinical trials [14], [15]. These results indicate involvement of other components, besides Hcy, as being key players in the disorders mentioned above.

Considering the remarkable cytoprotective action of physiological H_2_S and glutathione we posited that cancer cells might exploit this unique feature of CBS to produce H_2_S when under oxidative stress or upon cytotoxic insult. In this context, we focused on epithelial ovarian cancer, which is the leading cause of gynecologic cancer death in women. Most patients respond initially to platinum-based chemotherapy after surgical debulking, however relapse is very common and ultimately platinum resistance emerges. The mechanism of this recurrence and evolution of drug-resistance phenotype however remains poorly understood [16], [17]. To the best of our knowledge, this is the first report describing a role for CBS in maintaining cellular health of ovarian cancer cells by tuning cellular redox behaviour and mitochondrial energy production. Silencing CBS significantly inhibits ovarian cancer cell proliferation, metastatic nodule formation and sensitizes them to cisplatin both *in vitro* and in pre-clinical orthotopic mouse models *in vivo*. Mechanistically, silencing CBS severely reduces cellular GSH levels, impairs H_2_S production, activates tumor suppressors such as p53 and inhibits NF-κB activation. CBS co-localizes with mitochondrial markers in cancer cells, and silencing CBS decreases mitochondrial oxygen consumption with a concomitant increase in reactive oxygen species (ROS) production. These results together indicate that CBS plays an important role in regulating the redox balance and metabolism of the ovarian cancer cells promoting tumor growth and metastasis.

## Materials and Methods

### Ethics Statement

#### Patient samples

All 210 participants enrolled through 2009, from whom tissue for TMA was obtained, provided written informed consent for an IRB-approved protocol (09-008365) and clinical data was abstracted for all cases. All studies were approved by the Mayo Clinic Institutional Review Board (Protocol # 09-008365).

#### Animal experiments

Female athymic nude mice (NCr-nu) were purchased from the National Cancer Institute-Frederick Cancer Research and Development Center (Frederick, MD). All mice were housed and maintained under specific pathogen-free conditions in facilities approved by the American Association for Accreditation of Laboratory Animal Care (Acuf# 01-12-01531) and in accordance with current regulations and standards of the U.S. Department of Agriculture, U.S. Department of Health and Human Services, and NIH. All studies were approved by the University of Texas M. D. Anderson Cancer Center Institutional Animal Care and Use Committee.

### Reagents

Details of all reagents used are provided in *File S1.* OV167 and OV202 (obtained from V. Sridhar, Mayo Clinic) cell lines were grown in MEM and DMEM respectively supplemented with 10% FBS and 1% antibiotic (penicillin/streptomycin). OVCAR-5 was from ATCC and grown in DMEM with 10% fetal bovine serum and 1% antibiotic (penicillin/streptomycin). A2780 cells (Sigma-Aldrich) were grown in RPMI with 10% FBS and 1% antibiotic (Penicillin/Streptomycin) according to the provider's recommendation. OVCAR-5 cells were grown in DMEM high glucose with 10%FBS, L-Glutamine and NEAA. OSE (tsT) cells were grown in MCDB105 media with 15%FBS and 1% hygromycin. The A2780/CP-70 cell lines were grown according to our previously published procedures [18]. SKOV3 cell line from ATCC and SKOV3-ip from V. Sridhar’s laboratory (Mayo Clinic) were grown in McCoy’s 5A media supplemented with 10% FBS and 1% antibiotic.

### Study Participants and Construction of Tissue Microarrays (TMAs)

TMAs were created from formalin-fixed, paraffin-embedded tumors of 210 Mayo Clinic cases enrolled through December 2009. All participants provided written informed consent for an IRB-approved protocol (09-008365) and clinical data was abstracted for all cases. All studies were approved by the Mayo Clinic Institutional Review Board (Protocol # 09-008365).

We used an automated Beecher Instruments ATA-27 arrayer following pathologist review indicating tumor location. Three 0.6-mm cores were removed from each case paraffin block and placed in a recipient paraffin block according to a randomized electronic TMA map. Recipient blocks were sliced into 5-µm sections and mounted on charged slides.

### Antibody, Immunohistochemistry, and Scoring

TMA staining was performed on the Leica BOND auto stainer, using bond polymer refine detection kit (catalog #DS9800, Leica Microsystems). Slides were exposed to primary antibody recognizing CBS (1∶1000, polyclonal A01, Abnova), after optimizing staining conditions on positive control tissues (granulosa cell tumors). Negative controls included a nonspecific isotype match and negative mouse sera (1∶500); Slides were scored independently by 2 authors (R.B. and E.B.), and discrepancies were resolved by a gynecologic pathologist (*File S1*).

### Transfection and Knockdown

Cells were transfected with scrambled control siRNA or CBS siRNA (Hs_CBS_5 FlexiTube siRNA (SI02777159, QIAGEN)/SASI_Hs01_00214623 (Sigma)) by HiPerFect® transfection agent in suspension with a slight modification of the manufacturer’s protocol (*File S1*).

### Cell Lysis and Western Blotting

Cell lysis and western blotting was carried out as per already published procedure [19]. The primary antibodies were used at a concentration as follows: CBS (1∶1000 dilution), CSE antibody (1∶1000), α-tubulin (1∶2000), β-Actin (1∶1000 dilution), NF-κB p65 (1∶1000 dilution) and p53 antibody (1∶200 dilution).

### Realtime PCR

Total RNA was isolated from transfected cells using RNeasy Plus Mini kit (QIAGEN). RNA was first retrotranscribed using iScript cDNA Synthesis kit (Bio-Rad) and then realtime PCR was carried out using TaqMan® SYBR Green Master Mix (Applied Biosystems). The primers for human CBS (PPH13484B-200) and beta actin (PPH00073G-200 ACTB) were from QIAGEN. For CSE, custom designed primers (Forward: CAGCAATTACACCAGAAACCAAG and Reverse: CAGCCTTCAATGTCAATCACC) were used. The comparative C_t_ method was used to calculate the relative abundance of the mRNA and compared with that of beta actin expression [20]. The experiments were performed thrice in triplicate.

### Cell Proliferation Assay

Transfected ovarian cancer cells were collected by trypsinization, counted seeded in 24-wellplates (4×10^4^ per well)and cultured for 24 h and (3H)thymidine assay carried out as previously described [21] (*File S1*).

### Homocysteine (Hcy) Measurement

Hcy levels in the cell lysates of Sc-siRNA and CBS siRNA treated A2780 cells were determined 48 h after transfection by liquid chromatography tandem mass spectrometry (LC/MS/MS) (*File S1*).

### Homocysteine Overdose Experiment

Approximately, 10^4^ A2780, OSE or SKOV3-ip cells per well were plated in a 96 well plate with respective growth culture media. After 24 h, various concentrations of Hcy dissolved in growth culture media were added to the cells and incubated for 24 h in a CO_2_ incubator. 24 h post-treatment, the cell viability was assessed by MTS assay as mentioned above.

### H_2_S Measurement

H_2_S concentrations of untreated or AOAA treated ovarian cancer cells were measured following a literature reported protocol with minor modifications [22]. The H_2_S concentration of each sample was calculated against a calibration curve of Na_2_S.

### H_2_S Rescue Experiment

A2780 cells were transfected with scrambled control siRNA or CBS control siRNA as mentioned above. 48 h post-transfection, the cells were harvested by trypsinization and 10^4^ cells/well were plated in a 96 well plate. After 12 h, various concentrations of Na_2_S dissolved in RPMI-10%FBS were added to the cells and incubated for 24 h in a CO_2_ incubator. After 24 h, the cell viability was assessed by MTS assay as mentioned above.

### GSH Assay

The assay was performed according to manufacturer's protocol (Cayman Chemicals). Briefly, the ovarian cancer cell lines were transfected with the scrambled control or CBS siRNA in 60 mm culture plates as mentioned above. After 48 h, the cells were scraped off and collected in 50 mM MES pH 6.0 and lysed by sonication at 4°C. The lysate was then spun at 14000 rpm for 10 min at 4°C and the clear supernatant (i.e. cell lysate) was collected. The lysate was de-proteinated using the TEAM reagent and the total cellular GSH determined against a standard curve generated simultaneously by measuring absorbance at 405 nm for 25 min after addition of assay cocktail. The experiment was performed in triplicate and significance determined using two-sided Student's t test, P≤0.05 was considered significant.

### GSH Rescue Experiment

A2780 cells were transfected with scrambled control siRNA or CBS control siRNA as mentioned above. Post 48 h transfection, the cells were harvested by trypsinization and 10^4^ cells/well were plated in a 96 well plate. After 12 h, various concentrations of reduced glutathione dissolved in RPMI-10% FBS were added to the cells and incubated for 24 h in a CO_2_ incubator. Post 24 h treatment, the cell viability was assessed by MTS assay with Cell Titer 96® (Promega) as per the manufacturer’s protocol. The cell viability was expressed as a percentage ratio of the absorbance of the treated cells to the untreated controls.

### ROS Assay

The ROS assay was carried out in A2780 cells 48 h post-transfection with scrambled control or CBS siRNA after a modification of a previously published procedure using flow cytometry [23] (*File S1*).

### Luciferase Reporter Assay

Approximately, 2×10^4^ number of A2780 cells were plated per well of 96 well plate, the day before transfection. The following day the cells were transfected with a mixture of NF-κB driven firefly luciferase plasmid (100 ng), renilla luciferase plasmid (50 ng) and siRNA (200 nM) per well using Lipofectamine and Plus reagent (Invitrogen) according to manufacturer’s protocol. Post 48 h transfection, the culture media were discarded and were assayed for firefly luciferase activity and renilla luciferase activity using Dual Glo Luciferase Assay System (Promega) as per the manufacturer’s method. The ratio of firefly luciferase activity to renilla luciferase activity measured by luminescence was then calculated and the data were normalized. The experiment was performed in triplicate and significance determined using two-sided Student's t test, P≤0.05 was considered significant.

### Confocal Microscopy

Localization of CBS was determined by immunostaining followed by confocal microscopy. Approximately, 1.5×10^3^ cells were plated per chamber of a 4-chambered slides. After 12 h, the cells were treated with 200 nM Mitotracker Green (Invitrogen) for 30 min at 37°C in complete media fixed with 4% PFA, permeabilized with 0.1% TritonX-100 in PBS, blocked with 4% BSA in PBS, stained with primary CBS antibody (dilution 1∶100) in 1% BSA-PBS, blocked with 5% goat serum in 1% BSA-PBS and stained with Cy3-labelled goat anti-rabbit secondary antibody. The cells were washed 3×3 min with PBS after each step during the immunostaining. The images were acquired using Zeiss LSM510 microscope and processed using ImageJ (NIH).

### Oxygen Consumption Experiments

High-resolution respirometry (Oxygraph, Oroboros Instruments, Innsbruk, AT) was used to measure oxygen consumption rates in intact cells [24]. DatLab software (Oroboros Instruments, Innsbruk AT) was used to determine O_2_ flux under acute AOAA treatment and in siRNA treated A2780 cells. Oxygen flux rates were expressed per million of cells *(File S1).*

### Mitochondrial ROS Production

Mitochondrial ROS levels were determined in scrambled control siRNA and CBS siRNA treated cells 48 h post-transfection by MitoSOX (Invitrogen) staining was carried out as per literature protocol [25] *(Detailed in File S1)*.

### Citrate Synthase (CS) Activity

CS activity was measured spectrophotometrically in the whole cell lysates of A2780 cells after treatment with different doses of AOAA for 3 h following a literature reported method [26].

### NAD/NADH Ratio Measurement

NAD/NADH ratio was measured using Abcam NAD/NADH Assay Kit (ab65348) in whole cell lysates according to manufacturers’ protocol (details provided in *File S1*).

### Total ATP and ADP/ATP ratio

Total ATP levels in CBS silenced A2780 cells and AOAA treated A2780 cells were measured using Sigma Adenosine 5′-triphosphate (ATP) Bioluminescent Assay Kit (FLAA) and ADP/ATP ratio was measured using Abcam’s ADP/ATP ratio assay kit as per the manufacturers’ protocols. Detailed protocol in *File S1.*

### Liposomal siRNA Preparation

For *in vivo* delivery, siRNA was incorporated into DOPC as previously described [18]. For details see *File S1.*

### Orthotopic Model of Ovarian Cancer

Before injection tumor cells into the mice (8–12 weeks old), the cells were washed twice with PBS, detached by 0.1% cold EDTA, centrifuged for 7 min, and reconstituted in HBSS (Invitrogen). Cell viability was confirmed by trypan blue exclusion. Tumors were established by i.p. injection of 1.0×10^6^A2780/CP20 cells. Once established, this tumor model reflects the growth pattern of advanced ovarian cancer [27]. To assess the effects of siRNA therapy on tumor growth, treatment was initiated 1 wk after i.p. injection of tumor cells. The individuals who did the necropsies, tumor collections, and tissue processing were blinded to the treatment group assignments (File S1).

### Immunohistochemistry of Tumor Samples

OCT frozen tumor samples were sectioned and stained for H&E, Ki-67 (Mib-1, Dako, M7240) or CD31 (PECAM-1, SantaCruz, sc-1506-R) as previously described [28]. (File S1).

### Statistical Analysis

All results are displayed as mean+/−s.d. Statistical significance was determined using two-tailed Student's t test, and a value of P≤0.05 (*) was considered significant and P≤0.01 as highly significant (**). For comparison amongst multiple groups, One-way ANOVA was used (File S1).

## Results

### CBS is Overexpressed in Primary Epithelial Ovarian Cancer and Ovarian Cancer Cell Lines

**Figure 1:**
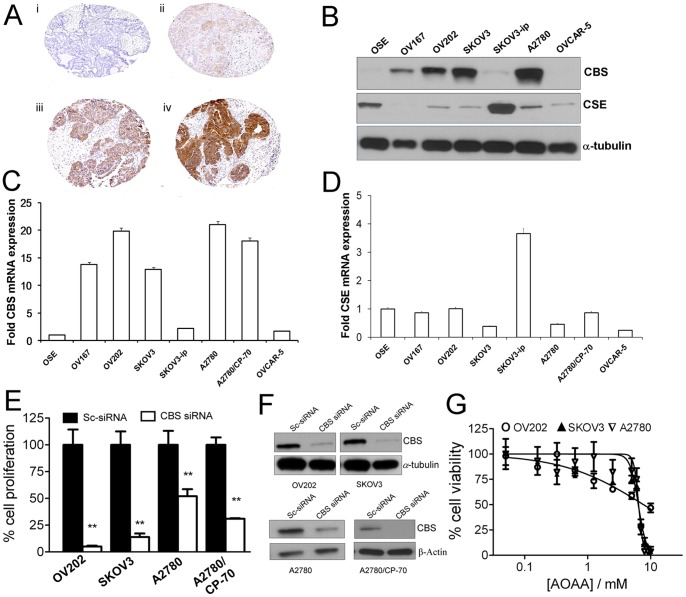
Expression and phenotypic effects of CBS *in vitro.* (A) Immunohistochemical staining of a tissue microarray of epithelial ovarian cancer samples. Representative images are shown of none (i), weak (ii), moderate (iii), and (iv) strong staining. (B) Expression of CBS and CSE in various ovarian cell lines as determined by immunoblotting. α-tubulin is used as the loading control. (C) RT-PCR data showing the expression of CBS mRNA in various ovarian cell lines. (D) RT-PCR data showing the expression of CSE mRNA in various ovarian cell lines. (E) Effect of CBS knockdown on the proliferation of OV202, SKOV3, A2780 and A2780/CP-70 cells and (F) Immunoblotting data to determine the extent of siRNA-mediated knockdown. (G) Effect of CBS inhibition on the proliferation of OV202, SKOV3 and A2780 cells by AOAA (24 h treatment) determined through MTS assay.

Tissue distribution studies in humans have revealed that the liver and pancreas have the highest expression of CBS mRNA with some expression in the brain and kidney [29]. In murine models, CBS has been suggested to be involved in oocyte development and *cbs* ^−/−^ mice suffer from uterine dysfunction [11], [30]. To understand the role of CBS in human ovarian cancer, we assessed the expression level of CBS in primary ovarian cancer specimens and its relationship to surgical-pathologic factors. Utilizing a series of tissue microarrays (TMAs) constructed from primary epithelial ovarian cancers we identified 210 cases with evaluable staining using TMAs. We found expression of CBS to be common in the cytosol of primary ovarian tumors, particularly in serous carcinoma, the most common histologic variant. Table 1 summarizes the demographic, clinical and histologic factors that were evaluated for an association with moderate to strong CBS expression (Table 1). Briefly, moderate-strong expression was associated with serous histology (69.8% vs. 41.0% serous vs. nonserous, p<0.001) and higher grade cancers (64.7% vs. 31.6%, grade 3 or 4 vs. grade 1 or 2, p = 0.005) (Fig. 1A). While statistically significantly different across stages, expression was still present in 35% of early stage tumors. When considering only the 149 serous cases in the larger cohort, expression was moderate to strong in 8/11 early stage (FIGO stages I and II) cancers, suggesting that CBS expression is a relatively early characteristic of serous ovarian cancers. Having demonstrated the expression of CBS in human tumor tissues, we compared the expression of CBS in normal ovarian vs. cancer cell lines. We employed quantitative real-time PCR (qRT-PCR) and immunoblotting to assess the expression of CBS at the mRNA and protein levels respectively (Fig. 1B–D). Both at the mRNA and the protein level, minimal to no expression of CBS was observed in the non-malignant ovarian surface epithelial cell line (OSE). However 4 out of 6 cancer cell lines expressed significantly high CBS both at the protein and the mRNA level. Interestingly, expression of CSE was observed in cell lines that expressed little to no CBS including OSE, suggesting an inverse correlation between expressions of the two enzymes (Fig. 1B). Having confirmed that CBS is expressed both in cell lines, and in primary ovarian cancers we next sought to examine the functional significance of CBS.

**Table 1:** Demographic, clinical and histologic factors that were evaluated for an association with moderate to strong CBS expression.

Characteristic	Moderate/StrongNo. (%)	Chi-squarep value
**Age at diagnosis (years)**		0.46
21–52 (N = 50)	27 (54.0%)	
53–60 (N = 55)	36 (65.5%)	
61–71 (N = 58)	34 (58.6%)	
72–87 (N = 47)	32 (68.1%)	
**Serous Histology**		<0.001
No (N = 61)	25 (41.0%)	
Yes (N = 149)	104 (69.8%)	
**Grade**		0.005
Not recorded (N = 21)	13	
1 or 2 (N = 19)	6 (31.6%)	
3 or 4 (N = 170)	110 (64.7%)	
**Stage**		0.002
Not recorded (N = 2)	1	
I or II (N = 40)	14 (35.0%)	
IIIa or IIIb (N = 9)	6 (66.7%)	
IIIc (N = 129)	89 (69.0%)	
IV (N = 30)	19 (63.3%)	
**Debulking**		0.09
Not recorded (N = 6)	2	
Optimal (N = 181)	109 (60.2%)	
sub-optimal (N = 23)	18 (78.3%)	

### Downregulation of CBS Impairs Cell Proliferation *in vitro*

To determine any role for CBS in ovarian cancer cell proliferation, CBS was silenced in A2780, A2780/CP-70, OV202 and SKOV3 cells using CBS-specific siRNA. To rule out non-specific targeting of the siRNA, knockdown and proliferation was assessed with siRNA from two different sources. A significant decrease in proliferation was observed in all ovarian cancer cell lines studied upon CBS knockdown by (^3^H)-thymidine incorporation (Fig. 1E). Efficient knockdown of CBS (∼80%) was also confirmed by immunoblotting 48 h post-transfection, as compared to scrambled siRNA transfected cells (Fig. 1F). To further confirm the effects of CBS silencing on ovarian cancer cell viability, we used a specific chemical inhibitor of CBS, Aminoxyacetic Acid (AOAA) [8]. Viability of A2780, OV202 and SKOV3 cells, treated with different concentrations of AOAA for 24 h were assessed by the MTS assay. Cell viability decreased sharply to 50% at ∼7 mM AOAA concentration with further dose-dependent decreases until at ∼10 mM concentration for A2780 and SKOV3 cells whereas in OV202 the effect was less pronounced (Fig. 1G). These data suggest that a threshold limit for CBS activity exists that is required to sustain proliferation of ovarian cancer cells.

### Downregulation of CBS Alters Antioxidant Levels, Triggers Apoptotic Cascades and Enhances Drug Sensitivity

**Figure 2:**
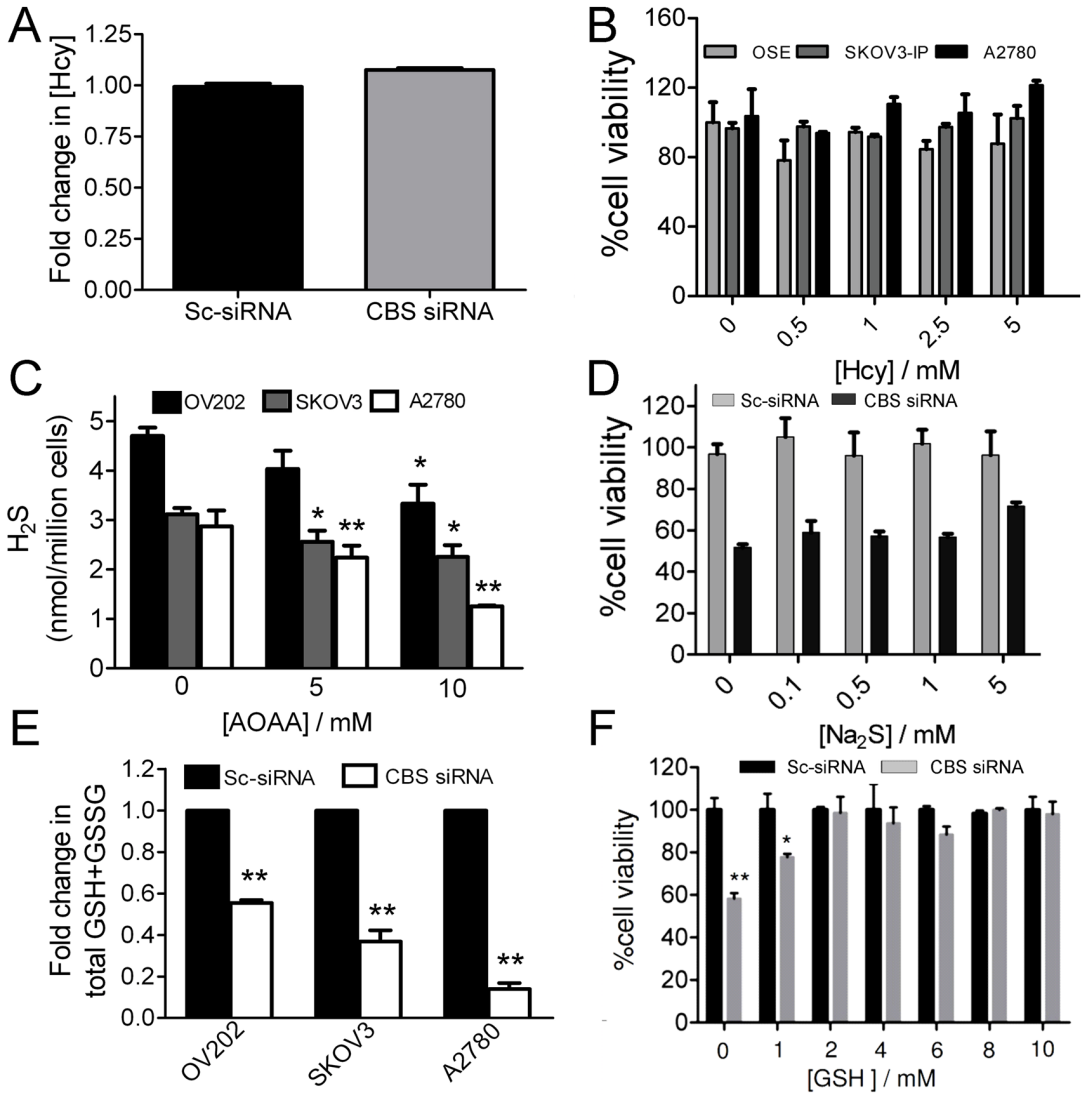
Effect of CBS knockdown on metabolite levels. (A) Change in Hcy levels in the cell lysates of Sc-siRNA and CBS siRNA treated A2780 cells measured by mass spectrometry. (B) Hcy overdose experiment to demonstrate the effect of Hcy (24 h treatment) on the proliferation of OSE, A2780 and SKOV3-ip cells by MTS assay. (C) H_2_S levels in OV202, SKOV3 and A2780 cells after chronic inhibition with different doses of AOAA for 3 h. (D) Na_2_S rescue experiment after knockdown of CBS in A2780 cells. The cells were treated with different doses of Na_2_S for 24 h and the cell viability was determined by MTS assay. (E) Change in total glutathione level 48 h post-transfection with scrambled control and CBS siRNA in OV202, SKOV3 and A2780 cells. (F) GSH rescue experiment after knockdown of CBS in A2780 cells. The cells were treated with various doses of GSH for 24 h and the cell viability was assessed by MTS assay.

The output of CBS inhibited reactions in the cell could be, a) a buildup of Hcy b) decreased H_2_S and c) decreased cystathionine, the precursor for GSH. Therefore we first tested if Hcy was a causal factor for the decreased proliferation (Fig. 2A,B). We supplemented the culture media with increasing concentrations of Hcy and then determined viability of OSE and the ovarian cancer cells (SKOV3-ip and A2780) using the MTS assay. Increasing Hcy levels had no impact on viability of any of the cells tested thus indicating that decreased levels of H_2_S or cystathionine were probably responsible for reduced proliferation (Fig. 2B). Also, no significant difference in cellular Hcy levels were observed as measured by mass spectrometry upon CBS knockdown in A2780 cells, further ruling out a role for Hcy in causing reduced proliferation (Fig. 2A). However, lack of significant differences in Hcy levels could be because Hcy can undergo quick conversion to methionine by methionine synthase or can be utilized by CSE to produce H_2_S but not cystathionine [31].

**Figure 3:**
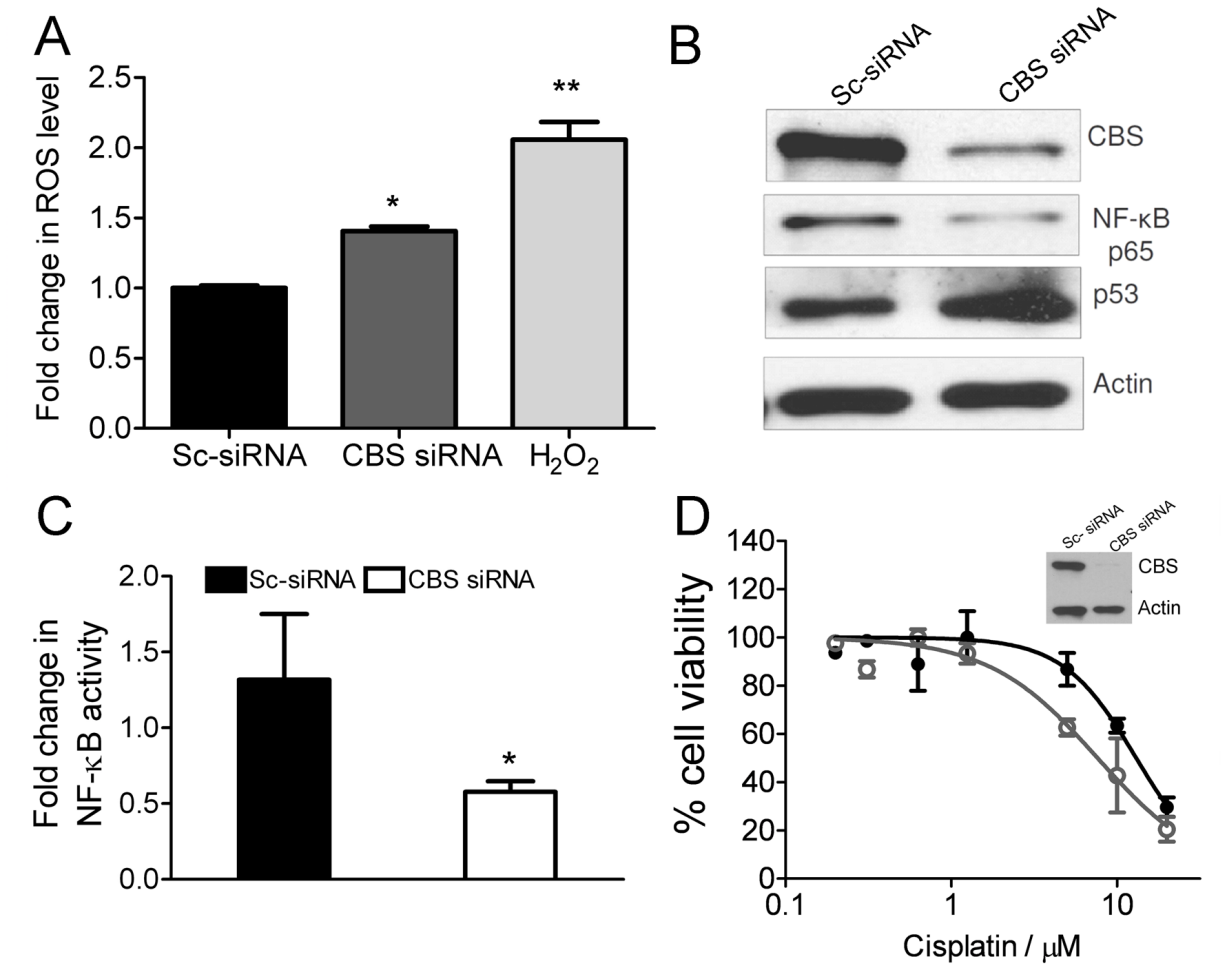
Silencing of CBS increases oxidative stress and sensitizes to Cisplatin chemotherapy. (A) Effect of CBS knockdown (48 h post-transfection) on total ROS level determined by DCF assay and quantified by flow cytometry. (B) Immunoblotting data exemplifying the effect of CBS silencing on the expression of p53 and NF-κB p65 in A2780 cells. β-actin serves as the loading control. (C) Luciferase reporter assay showing the effect of CBS knockdown on the activity of NF-κB in A2780 cells transfected with Firefly luciferase with multiple NF-κB binding site and scrambled control siRNA or CBS siRNA. *wt*-renilla luciferase was transfected to normalize firefly luciferase activity. (D) CBS silencing enhances the activity of cisplatin in A2780 cells. The (•) and (О) shows the effect of cisplatin on scrambled control siRNA and CBS siRNA treated cells. The cell viability was determined after treatment with increasing doses of cisplatin for 24 h through MTS assay and the cell viability was expressed as a percentage ratio of treated cells to the untreated controls. The IC_50_ values were obtained by non-linear regression analysis.

We next tested the contribution of H_2_S in supporting proliferation of ovarian cancer cell lines. A dose dependent decrease in intracellular levels of H_2_S was observed 3h after inhibition of CBS with different concentrations of AOAA (Fig. 2C). The overall decrease in H_2_S levels of OV202 cells was less pronounced than that in A2780 and SKOV3 cells which could be due to the existence of alternative H_2_S synthesis pathway in OV202 cells. These results indicate that CBS plays a critical role in H_2_S synthesis in these ovarian cancer cells. However, the role of CSE could not be ruled out at this point although it is unlikely since majority of the ovarian cancer cell lines tested here demonstrated a low to negligible expression of CSE both at the messenger and at the protein level. To further confirm a role of H_2_S we supplemented the culture media with increasing concentrations of an H_2_S donor, sodium sulphide (Na_2_S) [(32, 33] in scrambled siRNA and CBS siRNA treated A2780 cells. At 5 mM Na_2_S concentration a ∼20% rescue in cell viability was observed (Fig. 2D). Partial rescue of cell proliferation by H_2_S in CBS-silenced cells suggests that pathways other than H_2_S biosynthesis such as GSH might also play an important role. Therefore, we next focused on deciphering a role of GSH in ovarian cancer cell proliferation. Silencing CBS significantly decreased total cellular glutathione (GSH+GSSG) levels in ovarian cancer cells dramatically compared to the control cells (Fig. 2E). Importantly, a GSH concentration dependent rescue of cellular viability was observed in CBS silenced A2780 cells, with complete rescue at 2 mM concentration for 24 h (Fig. 2F). Simultaneously increase in cellular ROS levels were observed as determined by the H_2_carboxy DCF fluorescence assay in CBS-silenced A2780 cells (Fig. 3A) which corroborates with the fall in total intracellular glutathione in CBS-silenced cells. These results suggest that the most significant effect of silencing CBS in cancer cells is inhibition of the cellular antioxidant machinery. In this regard, two other molecules, p53 and NF-κB have been shown to be remarkably redox-sensitive [34]–[38]. Indeed, silencing CBS in A2780 cells induced expression of p53 and decreased expression of the *RelA*/p65 subunit of NF-κB (Fig. 3B). In accordance, the activation of NF-κB was also inhibited by nearly 50% in CBS silenced A2780 cells as determined by a promoter luciferase assay having multiple NF-κB (p65/p50) binding sites (Fig. 3C). However, a more direct role for CBS besides enhanced ROS in causing decreased NF-κB activation is also possible. Since intracellular glutathione and ROS were remarkably affected in CBS silenced cells, we next determined if combination with cisplatin would enhance therapeutic efficacy [39]. Indeed treatment of A2780 cells with cisplatin decreased the IC_50_ value from 13.1 µM in control siRNA transfected cells to 7.9 µM in CBS siRNA transfected cells as determined by MTS assay, 24 h post cisplatin treatment (Fig. 3D). These data indicate that CBS by acting through glutathione and H_2_S can provide a major survival advantage to cancer cells against cytotoxic insult.

### Silencing CBS Reduces Mitochondrial Respiration and Inhibits ATP Synthesis

**Figure 4:**
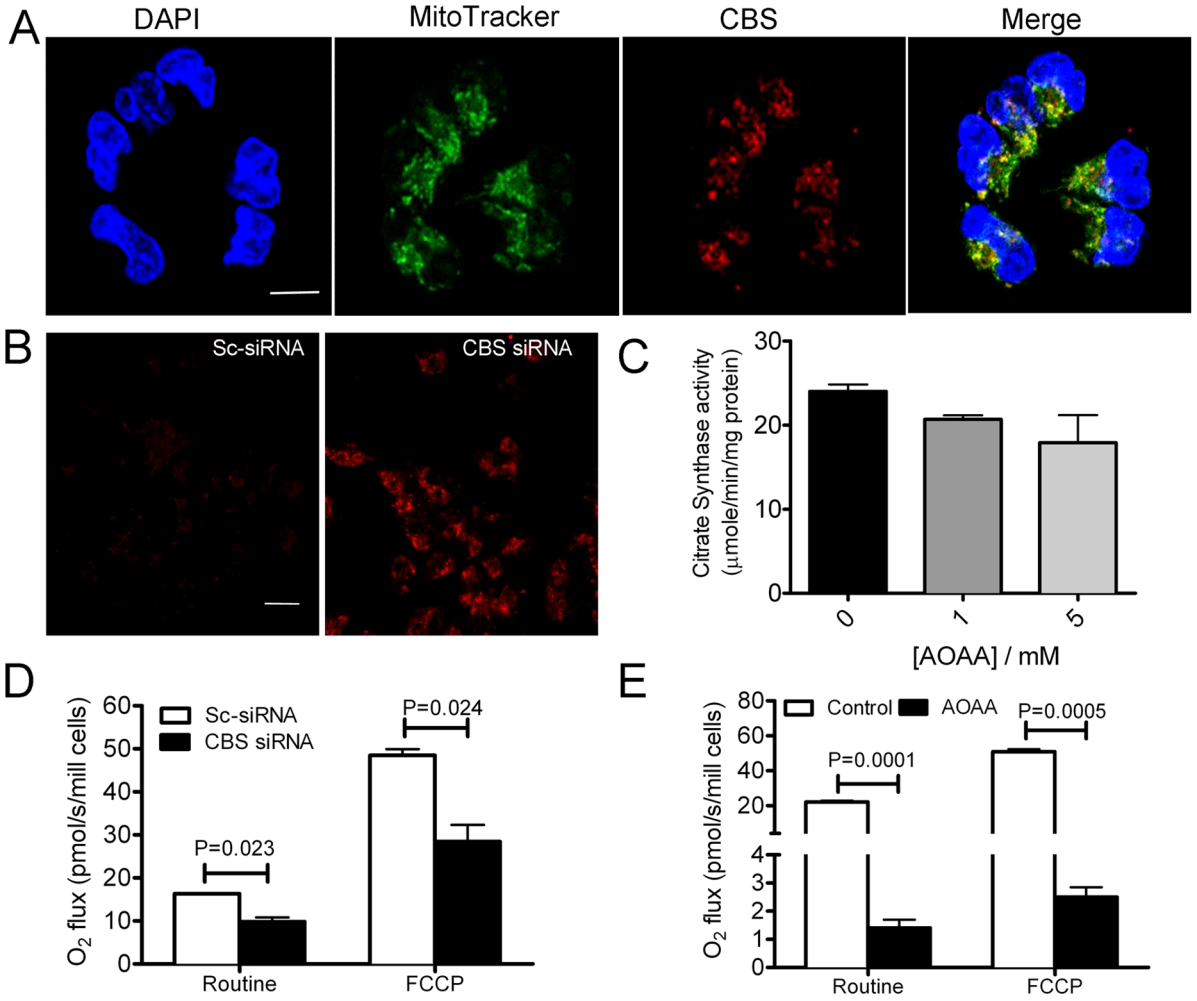
Localization and effect of silencing CBS on mitochondrial function. (A) Localization of CBS in A2780 cells determined by immunofluorescence using confocal microscopy. Nuclear stain with DAPI (blue channel), CBS (red channel), MitoTracker green (green channnel) was used to label mitochondria. Scale bar is 10 µm. (B) MitoSOX staining in live A2780 cells showing the buildup of mitochondrial superoxide upon silencing CBS. Scale bar is 30 µm. (C) Measurement of citrate synthase activity in AOAA-treated A2780 cells following 3 h of treatment. (D) Rate of oxygen consumption in scrambled control and CBS siRNA treated A2780 cells under resting condition and upon uncoupling with FCCP. (E) Rate of oxygen consumption in control (vehicle treated only) and 3 h AOAA treated A2780 cells under resting condition and upon uncoupling with FCCP.

In an effort to investigate a role of CBS besides amino acid metabolism, we first determined the cellular localization of the CBS enzyme using immunofluorescence. Punctate staining for CBS was observed in A2780 cells that almost completely co-localized with the mitochondria, as evidenced by MitoTracker, a mitochondrial probe (Fig. 4A). Interestingly, probing the expression in cytosolic and mitochondrial fraction revealed considerable expression of CBS in both the fractions under normoxic condition (data not shown). Given the mitochondrial distribution, we next probed mitochondrial ROS levels in CBS silenced cells using MitoSOX Red. In the presence of the superoxide anion, MitoSOX is oxidized and exhibits red fluorescence [25]. We observed a significant increase in fluorescence in CBS silenced A2780 cells compared to the scrambled control siRNA cells (Fig. 4B). These results suggest that a mitochondria specific increase in ROS ensue with CBS knockdown. To probe how ROS buildup affects mitochondrial function upon inhibiting CBS activity, we determined the activity of citrate synthase (CS) after chronic inhibition with AOAA. CS a key enzyme of the tricarboxylic acid (TCA) cycle is ubiquitous in all aerobic organisms and is an indicator of mitochondrial function [40]. A dose-dependent decrease in citrate synthase activity was noted with increasing concentrations of AOAA which confirmed that the ROS buildup is accompanied by decreased mitochondrial enzyme activity (Fig. 4C). Although the results were not statistically significant, such a trend, however, suggests inhibition of mitochondrial function upon CBS silencing. Since impaired electron flow across the ETC can increase ROS generation, these data corroborate our findings above.

To gain more insight into the role of CBS in mitochondrial bioenergetics, we studied the impact of silencing CBS upon mitochondrial respiration. Basal mitochondrial oxygen consumption was severely affected upon treatment with the CBS specific inhibitor, AOAA or upon CBS knockdown compared to the control cells (Fig. 4D,E). In the presence of the uncoupler, FCCP, oxygen consumption increased several fold in control but not in CBS silenced or AOAA treated cells. These results indicate that the reduction in basal and maximally-stimulated mitochondrial respiration could be due to an ROS-induced disruption of the cytochrome chain.

**Figure 5:**
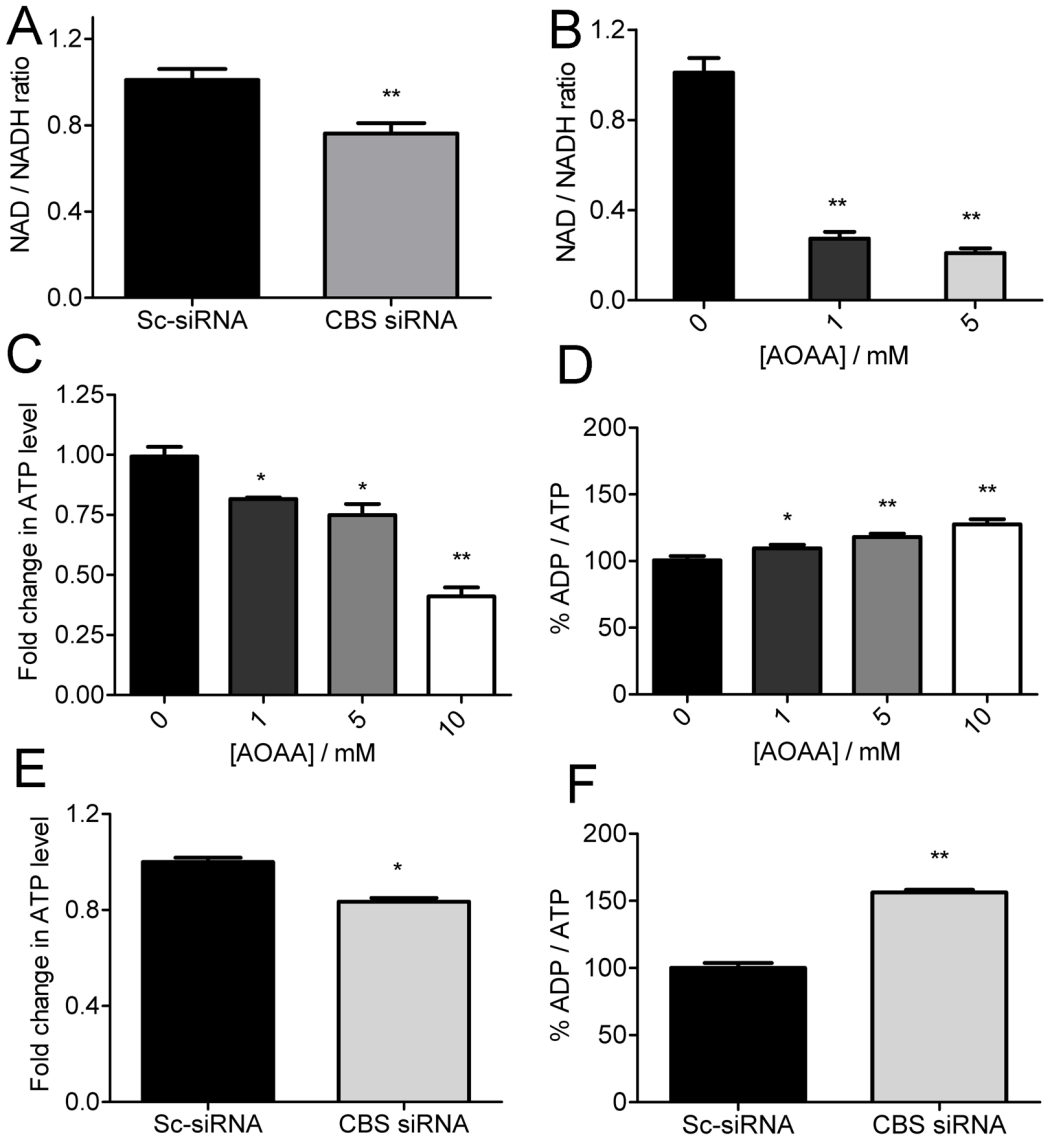
Effect of CBS silencing on OXPHOS. (A) Fold change in NAD/NADH ratio in CBS silenced A2780 cells measured 48 h after transfection (B) Fold change in NAD/NADH ratio in AOAA-treated A2780 cells measured after 3 h of treatment. (C) Fold change in total ATP levels in AOAA-treated A2780 cells with respect to scrambled control siRNA treated cells after 3 h treatment (D) Percent change in ADP/ATP ratio in AOAA-treated A2780 cells measured after 3 h of treatment. (E) Fold change in total ATP levels in CBS siRNA treated A2780 cells with respect to scrambled control siRNA treated cells measured 48 h after transfection (F) Percent change in ADP/ATP ratio in CBS siRNA treated A2780 cells with respect to scrambled control siRNA treated cells measured 48 h after transfection.

To obtain further insight into the impact of CBS silencing upon oxidative phosphorylation, we determined the NAD/NADH ratio and the end product of oxidative phosphorylation, *viz.* ATP (Fig. 5A–D). The NAD/NADH ratio significantly decreased upon downregulation of CBS either by siRNA or by inhibition with AOAA (Fig. 5A,B). The decrease is in agreement with the mitochondria-specific ROS stress which was observed as the NAD/NADH ratio is highly sensitive to the redox status of the cell [22]. Moreover, the diminished NAD/NADH ratio observed upon inhibition of CBS activity also exemplifies that mitochondrial ROS results from the impaired electron transport chain. To assess whether the change in NAD/NADH ratio affects the ATP synthesis, we measured the total ATP levels in A2780 cells after chronic inhibition of CBS with different doses of AOAA. AOAA treatment decreased the total ATP production in these cells and resulted in an increase in ADP/ATP ratio (Fig. 5C,D). Similar trend in reduction of ATP level was noted when CBS was silenced by siRNA, but the decrease in total ATP was not as marked as in case of AOAA treatment, still the results were statistically significant (Fig. 5E). Moreover the ADP/ATP ratio increased with silencing of CBS in the A2780 cells (Fig. 5F). These results reflect a potential role of CBS in supporting mitochondrial oxidative phosphorylation. In corroboration, a recent article has described a role for H_2_S in supporting ATP synthesis, under hypoxic stress, by translocation of CSE from the cytosol to the mitochondria in smooth muscle cells [41]. Our results, however, indicate that CBS supports ATP synthesis under resting conditions in ovarian cancer cells that could be mediated through H_2_S or GSH and clearly implicates a novel role for CBS in cancer cells rather than CSE which is marginally expressed.

### CBS Knockdown Attenuates Tumor Growth and Augments Cisplatin Activity *in vivo*

**Figure 6:**
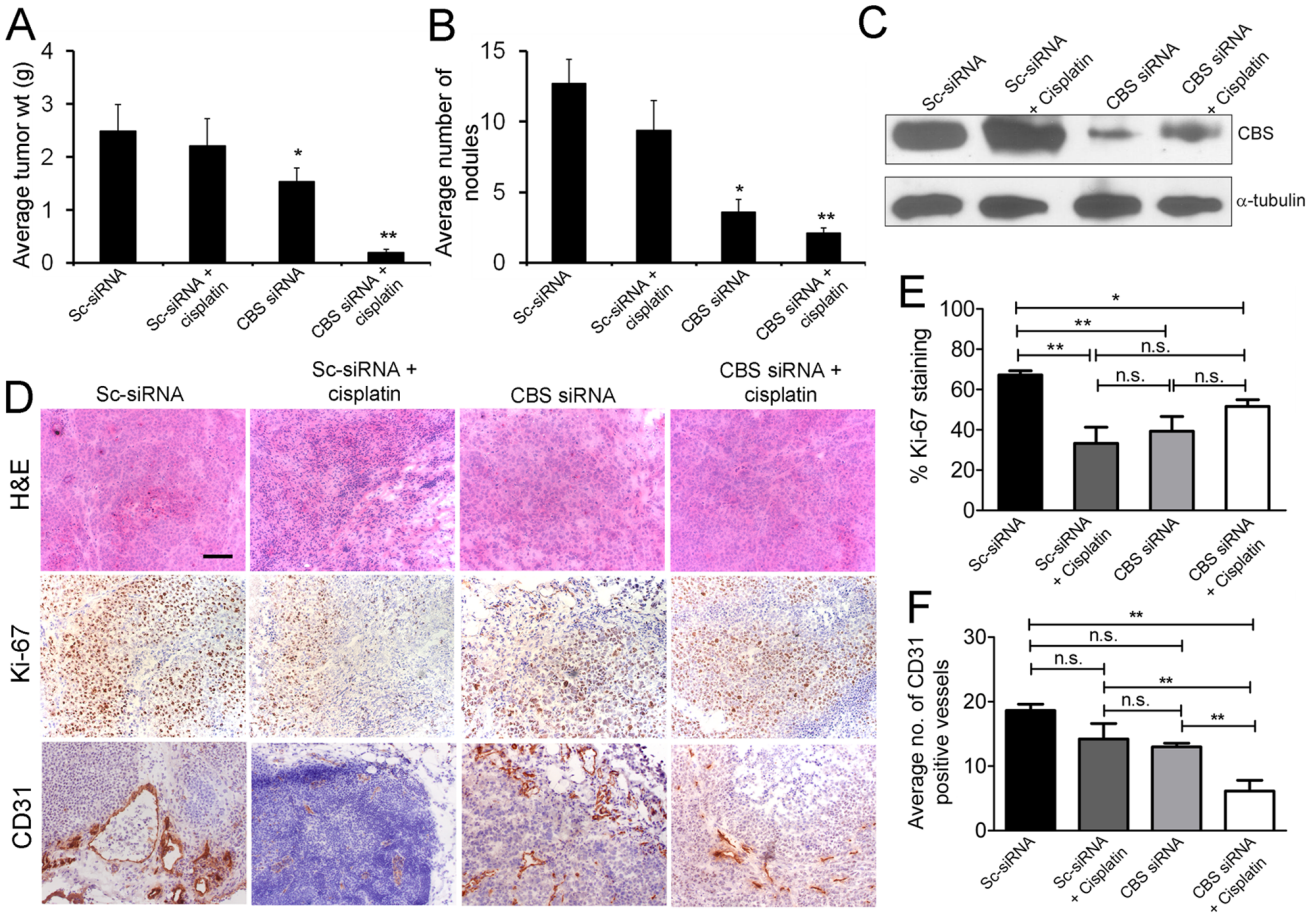
Effect of CBS knockdown on orthotopic chemoresistant ovarian cancer growth. (A)To assess the effects of siRNA therapy on tumor growth, treatment was initiated 1 wk after i.p. injection (1.0×10^6^ A2780/CP20) of tumor cells. Mice were divided into four groups (n = 10 mice per group): (i) control siRNA-DOPC (150 µg/kg i.p. twice weekly), (ii) control siRNA-DOPC (150 µg/kg i.p. twice weekly)+cisplatin (160 µg/mouse i.p. weekly), (iii) CBS siRNA-DOPC (150 µg/kg i.p. twice weekly), and (iv) CBS siRNA-DOPC (150 µg/kg i.p. twice weekly)+cisplatin ((160 µg/mouse i.p. weekly). Treatment was continued until 4 weeks after tumor inoculation before sacrifice. (A) Mouse and tumor weights and (B) the number of tumor nodules for each group were compared using Student's t test (for comparisons of two groups). A two-tailed P≤0.05 was deemed statistically significant. (C) Immunoblotting of tumor samples for confirmation of CBS knockdown. One animal from each group was selected for immunoblotting analysis. Lane 1 and lane 4 are from different blots. (D) Representative histology of tumors from mice xenografts of A2780/CP-20 cells with Ki67 expression (middle row) and CD31 expression (lower panel) acquired at 20X magnification. Scale bar represents 100 µm. (E) and (F) Quantification of Ki-67 staining and CD31 staining in the mouse xenografts respectively (n = 4). Statistical analysis was determined using One-way ANOVA with *P<0.05 and **P<0.01.

Given the remarkable effects of CBS *in vitro*, we next tested the effect of CBS knockdown in an orthotopic mouse model of ovarian cancer (A2780/CP-20). To simulate treatment of advanced small-volume disease, therapy was initiated a week after the injection of the tumor cells. A total of 40 mice were divided into four groups (n = 10 mice per group): (i) control siRNA-DOPC (150 µg/kg i.p. twice weekly), (ii) control siRNA-DOPC (150 µg/kg i.p. twice weekly)+cisplatin (160 µg/mouse i.p. weekly), (iii) CBS siRNA-DOPC (150 µg/kg i.p. twice weekly), and (iv) CBS siRNA-DOPC (150 µg/kg i.p. twice weekly)+cisplatin (160 µg/mouse i.p. weekly). After 4 weeks of therapy, the animals were sacrificed. A significant (∼40%) reduction in tumor weight was noted in the CBS siRNA treated group compared to the control siRNA treated group (Fig. 6A). Combination therapy of CBS siRNA and cisplatin resulted in a dramatic (∼90%) reduction in tumor weight compared to the cisplatin only treated group. To further corroborate this observation, the number of tumor nodules formed in each group was determined. A marked (∼70%) decrease in the number of tumor nodules was observed after treatment with CBS siRNA only and efficacy was further potentiated by combination therapy i.e. CBS siRNA and cisplatin, which showed a decrease of nearly 80% compared to the cisplatin only treated group (Fig. 6B). Knockdown of CBS was confirmed from immunoblotting data (Fig. 6C). Proliferative capacity of the cancer cells was also determined through Ki-67 staining, which showed significant reduction in the number of proliferating cancer cells with CBS knockdown alone (Fig. 6D, 6E). The number of vessels in the tumors was also determined through CD31 staining which exhibited a marked decrease with CBS knockdown alone or with the combination of CBS siRNA and cisplatin (Fig. 6D, 6F). These results further confirmed that CBS knockdown coupled to cisplatin treatment affects the proliferation of the cancer cells *in vivo* and substantially reduces the number of blood vessels thus resulting in attenuation of tumor growth. The outcome of these experiments signifies the potential of CBS as a therapeutic target in ovarian cancer, the inhibition of which would lead to disturbance in redox homeostasis and mitochondrial functioning.

## Discussion

The present study defines an important role for CBS in maintaining cancer cell health. Loss of CBS severely compromises cell viability that is exacerbated upon cisplatin treatment in cancer cells. Importantly both cisplatin sensitive and resistant cells are responsive to silencing of CBS. Over the past years, glutathione has emerged as a bonafide target in cancer cells [42]–[45]. However the enzymes in the GSH pathway, namely glutathione synthetase (GSS) and Gamma-glutamylcysteine synthetase (GCLM) are ubiquitous and therefore selective targeting of GSH in cancer cells has remained a major clinical challenge. In this regard, CBS represents an attractive target with minimal to no expression in OSE cells but with enhanced expression in cancer cells. Furthermore its role in mitochondrial energy metabolism by way of H_2_S generation might also be complementary in enhanced cancer cell killing. Recent studies in smooth muscle cells showed CSE as the only H_2_S generating enzyme present and upon stress it localizes to the mitochondria supporting ATP synthesis [41]. Our studies, however, show that ovarian cancer cells utilize CBS to support mitochondrial ATP production and at the same time protects against damage from leaky ETC by maintaining redox homeostasis. Also, a recent article by Szabo *et al*. have demonstrated that H_2_S produced by CBS in colon cancer cells plays an important role in bioenergetics thereby promoting tumor growth and proliferation and also promotes angiogenesis [46]. However the importance of CBS in maintenance of cellular redox homeostasis has not been discussed given the prominent role of CBS in cystathionine production, the precursor of cysteine.

In addition, the enhanced ROS generation observed in CBS silenced cells could be due to decreased scavenging activity owing to reduced GSH or due to reduced activity of enzymes such as catalase and superoxide dismutase (SOD). In fact a recent report demonstrated catalase and SOD augmenting activity of CBS like enzymes [8]. Enhanced ROS generation by itself can cause DNA damage and in combination with cisplatin this effect can be further compounded leading to sensitivity of cancer cells [47]. That silencing CBS reduces NF-κB activity is most likely through decreased GSH. In accordance a recent article demonstrated that the transcriptional activity of the p65 subunit was decreased upon GSH depletion [48]. Numerous studies in cancer cells including ovarian cancer have demonstrated activation of target survival genes by NF-κB and that inhibition of NF-κB increases the efficacy of cisplatin [49]. All of these studies corroborate our observations that silencing CBS enhances cisplatin efficacy. This is particularly relevant given our observations that CBS expression is common in primary serous ovarian cancer which are most commonly treated with platinum-based chemotherapy and for which platinum resistance is a major problem.

The master cell regulator p53 is remarkably sensitive to the cellular redox balance and is induced upon enhanced ROS as observed in CBS silenced cells. In fact, a microarray analysis of H_2_O_2_-treated human cells identified one-third of the 48 highly H_2_O_2_-reponsive genes as targets of p53 [50]. However, it is important to note that the decreased cell viability observed here was not dependent on p53 since it was observed in cells with wild-type or non-functional p53. All of these data support a protective/survival role for CBS in cancer cells. In accordance we demonstrated enhanced therapeutic efficacy of cisplatin in a chemoresistant orthotopic mouse model of ovarian cancer.

Importantly, previous studies have implicated Hcy as a causal factor in increased oxidative stress observed in *cbs* ^−/−^ mice [11]. However we demonstrate that GSH and H_2_S and not Hcy are the primary causal factors for oxidative stress observed in CBS silenced cancer cells. In addition CBS has a distinct function in mitochondrial health and thereby in cellular energetics. Taken together, CBS could be an excellent point of therapeutic intervention in ovarian cancer.

## Supporting Information

File S1 Detailed materials and methods used in the manuscript.
(DOCX)
