# Supplementary material for: Cystathionine Beta-Synthase (CBS) Contributes to Advanced Ovarian Cancer Progression and Drug Resistance
Source: PLoS One. 2013 Nov 13;8(11):e79167. doi: 10.1371/journal.pone.0079167 (PMC3827285; doi:10.1371/journal.pone.0079167)
Supplement: File S1 — Detailed materials and methods used in the manuscript. (DOCX) [file pone.0079167.s001.docx]

**Cystathionine beta-synthase (CBS) contributes to advanced ovarian cancer progression and drug resistance**

#### Supporting Materials and Methods

#### Materials and Methods

#### Reagents

#### Rabbit polyclonal CBS antibody (H-300, sc-67154), rabbit polyclonal CTH/CSE antibody (Proteintech, 12217-1-AP), mouse monoclonal p53 antibody (DO-1, sc-126) and rabbit polyclonal NF-κB (A) antibody (sc-109) were from Santa Cruz, CA, USA. Mouse monoclonal Beta-Actin antibody (A-2228) was from Sigma. Anti alpha-tubulin (ab4074) was from Abcam. siRNA against human CBS were from two sources (SI02777159 (QIAGEN) and (SASI_Hs01_00214623, Sigma) and scrambled control siRNA (1027280) was from QIAGEN, CA, USA. Cell culture media RPMI1640, DMEM and MEM were from Mediatech Inc, Manassas, VA, USA. Fetal Bovine Serum (FBS) was from Thermo Scientific. Antibiotic-Antimycotic solution, MEM Non-essential amino acids (NEAA), 0.25% Trypsin-EDTA and L-Glutamine solutions were from Gibco, NY, USA. HiPerFect^®^ was from QIAGEN. Lipofectamine, Plus reagent, Optimem-I and MitoSOX were from Invitrogen.

#### Cell Culture

#### OV167 and OV202 cell lines were grown in MEM and DMEM respectively supplemented with 10% FBS and 1% antibiotic (penicillin/streptomycin). OVCAR-5 was from ATCC and grown in DMEM with 10% fetal bovine serum and 1% antibiotic (penicillin/streptomycin). A2780 cells were grown in RPMI with 10% FBS and 1% antibiotic (Penicillin/Streptomycin) according to the provider's recommendation. OVCAR-5 cells were grown in DMEM high glucose with 10%FBS, L-Glutamine and NEAA. OSE (tsT) cells were grown in MCDB105 media with 15%FBS and 1% hygromycin. The A2780/CP-70 cell lines were grown according to our previously published procedures [1]. SKOV-3 cell line from ATCC and SKOV3-ip from V. Sridhar’s laboratory (Mayo Clinic) were grown in McCoy’s 5A media supplemented with 10% FBS and 1% antibiotic.

#### Study participants and construction of tissue microarrays (TMAs)

#### TMAs were created from formalin-fixed, paraffin-embedded tumors of 210 Mayo Clinic cases enrolled through December 2009. All participants provided written informed consent for an IRB-approved protocol and clinical data was abstracted for all cases. We used an automated Beecher Instruments ATA-27 arrayer following pathologist review indicating tumor location. Three 0.6-mm cores were removed from each case paraffin block and placed in a recipient paraffin block according to a randomized electronic TMA map. Recipient blocks were sliced into 5-μm sections and mounted on charged slides.

#### Antibody, immunohistochemistry, and scoring

#### TMA staining was performed on the Leica BOND auto stainer, using bond polymer refine detection kit (catalog #DS9800, Leica Microsystems). Slides were exposed to primary antibody recognizing CBS (1:1000, polyclonal A01, Abnova), after optimizing staining conditions on positive control tissues (granulosa cell tumors). Negative controls included a nonspecific isotype match and negative mouse sera (1:500); conditions were appropriate in that no background staining was observed without the specific primary antibody. Slides were prepared according to the manufacturer's instructions (BOND III, the Leica BOND auto stainer), and digital images were created as described previously (Lingle WA, 2005). Each tissue microarray slide was scanned using the Bacus Laboratories Inc. Slide Scanner (Bacus Laboratories, Inc.).  The BLISS system is able to digitally capture images at 480 x 752 pixel resolution and at 20x magnification.  Each core is composed of multiple tiles to create a mosaic or composite picture.  These tiled images were viewed using Microsoft Access via the Bacus Laboratories Active X component.  For the anti-CBS stained slides, the percentage of positive tumor and IHC staining intensity values for each core were collected.  After viewing all of the cores, the data was exported from Microsoft Access to an Excel spreadsheet where it was combined with clinical data for further statistical analysis. Extent was defined as the percentage of tumor cells staining positive for CBS protein (negative, 0%–10% of cells expressing; positive, >10% of cells expressing), and intensity was defined as the strength of antibody staining in tumor cells (none, weak, moderate and strong). Slides were scored independently by 2 authors (R.B. and E.B.), and discrepancies were resolved by a gynecologic pathologist. Intensity measures for each core were combined for analysis as negative (weak/none), and positive (moderate/strong).

#### Transfection and knockdown

#### Cells were transfected with scrambled control siRNA or CBS siRNA (Hs_CBS_5 FlexiTube siRNA (SI02777159, QIAGEN)/ SASI_Hs01_00214623 (Sigma)) by HiPerFect^®^ transfection agent in suspension with a slight modification of the manufacturer’s protocol. Briefly, 3 x 10^5^ cells were plated in 2 ml of RPMI1640 supplemented with 10% FBS and 1% antibiotic on a 60 mm culture dish and incubated for 10 min in a CO­_2_ incubator. During this incubation period, the transfection mixture was prepared by adding 20 µl of Hiperfect^®^ and 20 µl of 20 µM scrambled siRNA or CBS siRNA to 500 µl of Optimem-I and incubated for 10 mins at RT. After the 10 min incubation period, the transfection mixture was added to the cells in 60 mm dish and incubated for 48 h before any analysis.

#### Cell Lysis and Western Blotting

#### Cell lysis and western blotting was carried out as per already published procedure [2]. Briefly, harvested ovarian cancer cells, both treated and non-treated, were washed twice with ice-cold PBS and lysed in ice-cold radioimmunoprecipitation (RIPA) buffer with freshly added 0.01% protease inhibitor cocktail (Sigma) and incubated on ice for 30 min with occasional shaking. The cells along with the lysate were then scraped off using a cell scrapper, centrifuged at 14000 rpm for 10 min at 4°C, and protein estimation in the supernatant was carried out using BCA assay and 10 µg of protein was separated on 10% SDS-PAGE. The separated proteins were transferred on to a PVDF membrane, blocked with 10% milk in TBST for 1 h at RT, incubated in diluted primary antibody in 3% milk in TBST overnight at 4°C, washed 3X with TBST for 10 min each at RT and stained with HRP-conjugated secondary antibody (1:7500 dilution) in TBST for 1 h at RT. After 3X wash with TBST, the proteins were detected using luminol reaction. The primary antibodies were used at a concentration as follows: CBS (1:1000 dilution), CSE antibody (1:1000), α-tubulin (1:2000), β-Actin (1:1000 dilution), NF-κB p65 (1:1000 dilution) and p53 antibody (1:200 dilution).

#### Realtime PCR

#### Total RNA was isolated from transfected cells using RNeasy Plus Mini kit (QIAGEN). RNA was first retrotranscribed using iScript cDNA Synthesis kit (Bio-Rad) and then realtime PCR was carried out using TaqMan^®^ SYBR Green Master Mix (Applied Biosystems). The primers for human CBS (PPH13484B-200) and beta actin (PPH00073G-200 ACTB) were from QIAGEN. For CSE, custom designed primers (Forward: CAGCAATTACACCAGAAACCAAG and Reverse: CAGCCTTCAATGTCAATCACC) were used. The comparative C_t_ method was used to calculate the relative abundance of the mRNA and compared with that of beta actin expression [3]. The experiments were performed thrice in triplicate.

#### Cell Proliferation Assay

#### Transfected ovarian cancer cells were collected by trypsinization, counted seeded in 24-wellplates (4 × 10^4^ per well) and cultured for 24 h. Subsequently, 1 μCi of [^3^H]thymidine per mL of culture media was added to each well; 4 h later, cells were washed with chilled PBS, fixed with 100% cold methanol, and collected for measurement of trichloroacetic acid–precipitable radioactivity. Experiments were repeated at least three times each time in triplicate, and assay done as previously described [4].

#### Homocysteine (Hcy) Measurement

#### Hcy levels in the cell lysates of Sc-siRNA and CBS siRNA treated A2780 cells were determined 48 h after transfection. The cells were then lysed with PBS containing protease inhibitor cocktail. Homocystine, free plus bound as disulfide to proteins in the lysate, was reduced to homocysteine (Hcy) using Dithiothreitol (DTT) at room temperature. An internal standard containing d8-Homocystine was added which participates in the reduction, yielding an addition of 40 µM d4-Homocysteine (d4-Hcy). Proteins from the samples were then precipitated following reduction by the addition of acetonitrile containing formic acid and trifluoroacetic acid, to maintain reducing conditions and stabilize the solution for instrumental analysis by liquid chromatography tandem mass spectrometry (LC/MS/MS). The specific transitions for m/z 136 to m/z 90 and m/z 140 to m/z 94 for Hcy and d4-Hcy respectively were monitored.

#### Homocysteine Overdose Experiment

#### Approximately, 10^4^ A2780, OSE or SKOV3-ip cells per well were plated in a 96 well plate with respective growth culture media. After 24 h, various concentrations of Hcy dissolved in growth culture media were added to the cells and incubated for 24 h in a CO_2_ incubator. 24 h post-treatment, the cell viability was assessed by MTS assay as mentioned above.

#### H_2_S measurement

#### H_2_S concentrations of untreated or AOAA treated ovarian cancer cells were measured following a literature reported protocol with minor modifications [5]. Briefly, 6 x 10^6^ A2780 cells treated with AOAA (0-10 mM) were collected by trypsinization and resuspended in a total volume of 100 μL of the phosphate-buffered saline (pH 7.4) was then transferred directly into a tube containing zinc acetate (1% wt/vol, 187.5 μL) and NaOH (12%, 12.5 μL) to trap the H_2_S for 15 min at room temperature. The reaction was terminated by adding 1 mL of H_2_O (pH 12.8), 200 μL of N,N-dimethyl-p-phenylenediamine sulfate (20 mM in 7.2 M HCl), and 200 μL of FeCl_3_ (30 mM in 1.2 M HCl). The mixture was incubated at RT in dark for 15 min and finally 600 μL of the mixture was added to a tube with 150 μL of trichloroacetic acid (10% wt/vol) to precipitate protein. The precipitated protein was removed by centrifugation at 10,000 × g for 5 min, and absorbance at 670 nm of the resulting supernatant (200 μL) was determined using a 96-well microplate reader. The H_2_S concentration of each sample was calculated against a calibration curve of Na_2_S.

#### H_2_S Rescue Experiment

#### A2780 cells were transfected with scrambled control siRNA or CBS control siRNA as mentioned above. 48 h post-transfection, the cells were harvested by trypsinization and 10^4^ cells/well were plated in a 96 well plate. After 12 h, various concentrations of Na_2_S dissolved in RPMI-10%FBS were added to the cells and incubated for 24 h in a CO_2_ incubator. After 24 h, the cell viability was assessed by MTS assay as mentioned above.

#### GSH Assay

#### The assay was performed according to manufacturer's protocol (Cayman Chemicals). Briefly, the ovarian cancer cell lines were transfected with the scrambled control or CBS siRNA in 60 mm culture plates as mentioned above. After 48 h, the cells were scraped off and collected in 50 mM MES pH 6.0 and lysed by sonication at 4°C. The lysate was then spun at 14000 rpm for 10 min at 4°C and the clear supernatant (i.e. cell lysate) was collected. The lysate was de-proteinated using the TEAM reagent and the total cellular GSH determined against a standard curve generated simultaneously by measuring absorbance at 405 nm for 25 min after addition of assay cocktail. The experiment was performed in triplicate and significance determined using two-sided Student's t test, P≤0.05 was considered significant.

#### GSH Rescue Experiment

#### A2780 cells were transfected with scrambled control siRNA or CBS control siRNA as mentioned above. Post 48 h transfection, the cells were harvested by trypsinization and 10^4^ cells/well were plated in a 96 well plate. After 12 h, various concentrations of reduced glutathione dissolved in RPMI-10% FBS were added to the cells and incubated for 24 h in a CO_2_ incubator. Post 24 h treatment, the cell viability was assessed by MTS assay with Cell Titer 96^®^ (Promega) as per the manufacturer’s protocol. The cell viability was expressed as a percentage ratio of the absorbance of the treated cells to the untreated controls.

**ROS Assay**

The ROS assay was carried out in A2780 cells 48 h post-transfection with scrambled control or CBS siRNA after a modification of a previously published procedure [6]. The transfected cells were washed twice with pre-warmed PBS and incubated with 5 µM carboxy-H_2_DCFDA (Invitrogen, Carlsbad, CA) in fresh phenol red-free RPMI1640 for 30 min at 37°C. After the incubation, the excessive probe was washed off with 3X PBS washes. As a positive control, untransfected cells were treated with 0.03% H_2_O_2_ in phenol red-free RPMI1640 for 25 mins at 37°C and after 2X PBS washes probed with carboxy-H_2_DCFDA as above. The cells were harvested with 0.05% Trypsin-EDTA and transferred to FACS tubes in 400 µl phenol red-free RPMI1640-10% FBS. DCF fluorescence was quantified by flow cytometry (3 x 10^4^ cells) at an excitation wavelength of 488 nm and emission wavelength of 530 nm in a BD FACS Calibur flow cytometer.

#### Luciferase Reporter Assay

#### Approximately, 2 x 10^4^ number of A2780 cells were plated per well of 96 well plate, the day before transfection. The following day the cells were transfected with a mixture of NF-κB driven firefly luciferase plasmid (100 ng), renilla luciferase plasmid (50 ng) and siRNA (200 nM) per well using Lipofectamine and Plus reagent (Invitrogen) according to manufacturer’s protocol. Post 48 h transfection, the culture media were discarded and were assayed for firefly luciferase activity and renilla luciferase activity using Dual Glo Luciferase Assay System (Promega) as per the manufacturer’s method. The ratio of firefly luciferase activity to renilla luciferase activity measured by luminescence was then calculated and the data were normalized. The experiment was performed in triplicate and significance determined using two-sided Student's t test, P≤0.05 was considered significant.

#### Confocal Microscopy

#### Localization of CBS was determined by immunostaining followed by confocal microscopy. Approximately, 1.5 x 10^3^ cells were plated per chamber of a 4-chambered slides. After 12 h, the cells were treated with 200 nM Mitotracker Green (Invitrogen) for 30 min at 37 ºC in complete media fixed with 4% PFA, permeabilized with 0.1% TritonX-100 in PBS, blocked with 4% BSA in PBS, stained with primary CBS antibody (dilution 1:100) in 1% BSA-PBS, blocked with 5% goat serum in 1% BSA-PBS and stained with Cy3-labelled goat anti-rabbit secondary antibody. The cells were washed 3 x 3 min with PBS after each step during the immunostaining. The images were acquired using Zeiss LSM510 microscope and processed using ImageJ (NIH).

#### Oxygen Consumption Experiments

#### High-resolution respirometry (Oxygraph, Oroboros Instruments, Innsbruk, AT) was used to measure oxygen consumption rates in intact cells [7]. Experiments were performed in a 2ml chamber with continuous electromagnetic stirring and temperature maintained at 37 °C with a Peltier thermostat. Following equilibration with room air, intact cells were incubated at a density of 5-6 million cells in 2 ml culture media. Oxygen consumption was measured during routine respiration until steady-state O_2_ flux was reached. Following routine respiration, Trifluorocarbonylcyanide Phenylhydrazone (FCCP) was titrated in 1 μM steps to uncouple electron transport and stimulate mitochondrial respiration. The stepwise FCCP titration continued until respiration became inhibited. DatLab software (Oroboros Instruments, Innsbruk AT) was used to determine O_2_ flux under the 2 conditions. Oxygen flux rates were expressed per million of cells.

#### For scrambled control siRNA and CBS siRNA treated A2780 cells, approximately 3 x 10^6^ cells were collected by trypsinization 48 h post-transfection, counted and resuspended in 2 ml RPMI1640 supplemented with 10% FBS.

#### To determine the effect of acute treatment with CBS specific inhibitor AOAA on oxygen consumption, 3 x 10^6^ A2780 cells were taken in suspension after trypsinization in 2 ml RPMI1640 supplemented with 10% FBS, treated with AOAA (dissolved in RPMI1640-10% FBS) in the respirometry chamber to give a final concentration of 10 mM. pH matched RPMI1640-10% FBS was added as a control to negate the effect of decrease in pH which occurs upon dissolution of AOAA in RPMI1640-10% FBS. The effect of 3 h treatment with AOAA was also determined on A2780 cells. 3 x 10^6^ A2780 cells in 150 mm tissue culture dishes were treated with AOAA (Final: 10 mM) for 3 h at 37 °C, washed with PBS, trypsinized and resuspended in 2 mL of AOAA-treatment (Final: 10 mM) media and the oxygen consumption rates were determined with appropriate pH matched controls.

#### Mitochondrial ROS Production

#### Mitochondrial ROS levels were determined in scrambled control siRNA and CBS siRNA treated cells 48 h post-transfection by MitoSOX (Invitrogen) staining was carried out as per literature protocol [8]. Briefly, 48 h post-transfection the cells were treated with MitoSOX (Final: 5 µM) in serum-free RPMI 1640 for 10 min at 37 ºC, washed with warm serum-free RPMI 1640 and imaged with a Zeiss LSM510 laser scanning microscope with excitation wavelength of 543 nm and emission was collected with band pass filter of 560-615 nm.

#### Citrate Synthase (CS) Activity

#### CS activity was measured spectrophotometrically in the whole cell lysates of A2780 cells after treatment with different doses of AOAA for 3 h following a literature reported method [9].

#### NAD/NADH Ratio Measurement

#### NAD/NADH ratio was measured using Abcam NAD/NADH Assay Kit (ab65348) in whole cell lysates according to manufacturers’ protocol. For assessing in siRNA transfected cells, A2780 cells were transfected with siRNAs as mentioned above and after 48 h the assay was carried out as per the protocol. For measurement of NAD/NADH ratio after AOAA treatment, 3 x10^5^ A2780 cells were plated in 6-well plates and after 24 h treated with different doses of AOAA with pH matched controls for 3 h. After 3 h, the cells were processed as per the protocol.

#### Total ATP and ADP/ATP ratio

#### Total ATP levels in CBS silenced A2780 cells and AOAA treated A2780 cells were measured using Sigma Adenosine 5′-triphosphate (ATP) Bioluminescent Assay Kit (FLAA) as per the manufacturers’ protocol. For assessing ATP level in transfected cells, A2780 cells transfected with siRNAs as mentioned above and after 48 h the assay was carried out as per the protocol. For measurement of ADP/ATP ratio after AOAA treatment, 3 x10^5^ A2780 cells were plated in 6-well plates and after 24 h treated with different doses of AOAA with pH matched controls for 3 h. After 3 h, the cells were processed and the assay was carried out using ADP/ATP Ratio Assay Kit (Abcam) following the protocol therein.

#### Liposomal siRNA Preparation

#### For *in vivo* delivery, siRNA was incorporated into DOPC as previously described [1]. Briefly, siRNA and DOPC were mixed at a ratio of 1:10 (w/w) siRNA/DOPC in excess tertiary butanol. Tween 20 was added to the mixture at the ratio of 1:19 (Tween 20:siRNA/DOPC). After vortexing, the mixture was frozen in an acetone/dry ice bath and lyophilized. Before in vivo administration, this mixture was hydrated with 0.9% saline to a concentration of 25 µg/mL and 200 µL of mixture were used per injection.

#### Orthotopic Model of Ovarian Cancer

#### Female athymic nude mice (NCr-nu) were purchased from the National Cancer Institute-Frederick Cancer Research and Development Center (Frederick, MD). All mice were housed and maintained under specific pathogen-free conditions in facilities approved by the American Association for Accreditation of Laboratory Animal Care (Acuf# 12-02-18233) and in accordance with current regulations and standards of the U.S. Department of Agriculture, U.S. Department of Health and Human Services, and NIH. All studies were approved and supervised by the University of Texas M. D. Anderson Cancer Center Institutional Animal Care and Use Committee. All mice were used in these experiments when they were 8 to 12 wk old.

#### Before injection, tumor cells were washed twice with PBS, detached by 0.1% cold EDTA, centrifuged for 7 min, and reconstituted in HBSS (Invitrogen). Cell viability was confirmed by trypan blue exclusion. Tumors were established by i.p. injection of either 1.0×10^6^ CP20 cells. Once established, this tumor model reflects the growth pattern of advanced ovarian cancer [10].

#### To assess the effects of siRNA therapy on tumor growth, treatment was initiated 1 wk after i.p. injection of tumor cells. Mice were divided into four groups (n = 10 mice per group): (a) control siRNA-DOPC (150 µg/kg i.p. twice weekly), (b) control siRNA-DOPC (150 µg/kg i.p. twice weekly) + cisplatin (160 µg/mouse i.p. weekly), (c) CBS siRNA-DOPC (150 µg/kg i.p. twice weekly), and (d) CBS siRNA-DOPC (150 µg/kg i.p. twice weekly) + cisplatin (160 µg/mouse i.p. weekly). Treatment was continued until 4 weeks after tumor inoculation. At the time of sacrifice, mouse weight, tumor weight, number of nodules, and distribution of tumors were recorded. Tissue samples were snap frozen for lysate preparation or fixed in formalin for paraffin embedding. The individuals who did the necropsies, tumor collections, and tissue processing were blinded to the treatment group assignments.

#### Immunohistochemistry of tumor samples

#### OCT frozen tumor samples were sectioned and stained for H&E, Ki-67 (Mib-1, Dako, M7240) or CD31 (PECAM-1, SantaCruz, sc-1506-R) as previously described [11]. Quantification of Ki-67 was carried out by the percentage of Ki-67-positive cells in 4 fields at 20X maginification and scored using custom-written script in KS-400 software. Number of CD31 positive vessels were counted manually for 4 different fields at 20X magnification and the average number of vessels per treatment group were analyzed by One-way ANOVA.

#### Statistical Analysis

#### All results are displayed as mean+/-s.d. Statistical significance was determined using two-tailed Student's t test, and a value of P≤0.05 (*) was considered significant and P≤0.01 as highly significant (**). For comparison amongst multiple groups, One-way ANOVA was used.

#### For animal experiments, 10 mice were assigned per treatment group. To judge the necessary sample size for proposed experiments, we considered a two-way ANOVA model. For an effect size (ratio of fixed effect and residual SD) of 1.3, this sample size will be sufficient to provide 80% power for a test at significance level of 0.05. Mouse and tumor weights and the number of tumor nodules for each group were compared using Student's t test (for comparisons of two groups). Statistical analyses were done using Statistical Package for the Social Sciences 12.0 for Windows (SPSS, Inc.). A P value of ≤0.05 was deemed statistically significant.

For statistical analyses of patient samples, the chi-square test was used to evaluate the association between patient characteristics and moderate to strong CBS expression.

**References**

#### 1. Mangala LS, Zuzel V, Schmandt R, Leshane ES, Halder JB, et al. Therapeutic Targeting of ATP7B in Ovarian Carcinoma. *Clin Cancer Res.* 2009;15(11):3770-80.

#### 2. Bhattacharya R, Kwon J, Ali B, Wang E, Patra S, et al. Role of hedgehog signaling in ovarian cancer. *Clin Cancer Res.* 2008;14(23):7659-66.

#### 3. Schmittgen TD, Livak KJ. Analyzing real-time PCR data by the comparative C(T) method. *Nat Protoc.* 2008;3(6):1101-8.

#### 4. Mukherjee P, Bhattacharya R, Wang P, Wang L, Basu S, et al. Antiangiogenic properties of gold nanoparticles. *Clin Cancer Res.* 2005;11(9):3530-4.

#### 5. Hung YP, Albeck JG, Tantama M, Yellen G. Imaging cytosolic NADH-NAD(+) redox state with a genetically encoded fluorescent biosensor. *Cell Metab.* 2011;14(4):545-54.

#### 6. Chompoosor A, Saha K, Ghosh PS, Macarthy DJ, Miranda OR, et al. The role of surface functionality on acute cytotoxicity, ROS generation and DNA damage by cationic gold nanoparticles. *Small.* 2010;6(20):2246-9.

#### 7. Hutter E, Renner K, Pfister G, Stockl P, Jansen-Durr P, et al. Senescence-associated changes in respiration and oxidative phosphorylation in primary human fibroblasts. *Biochem J.* 2004;380(Pt 3):919-28.

#### 8. Mukhopadhyay P, Rajesh M, Hasko G, Hawkins BJ, Madesh M, et al. Simultaneous detection of apoptosis and mitochondrial superoxide production in live cells by flow cytometry and confocal microscopy. *Nat Protoc.* 2007;2(9):2295-301.

#### 9. Rooyackers OE, Adey DB, Ades PA, Nair KS. Effect of age on in vivo rates of mitochondrial protein synthesis in human skeletal muscle. *Proc Natl Acad Sci U S A.* 1996;93(26):15364-9.

#### 10. Merritt WM, Lin YG, Spannuth WA, Fletcher MS, Kamat AA, et al. Effect of interleukin-8 gene silencing with liposome-encapsulated small interfering RNA on ovarian cancer cell growth. *J Natl Cancer Inst.* 2008;100(5):359-72.

#### 11. Olive KP, Jacobetz MA, Davidson CJ, Gopinathan A, McIntyre D, et al. Inhibition of Hedgehog signaling enhances delivery of chemotherapy in a mouse model of pancreatic cancer. *Science.* 2009;324(5933):1457-61.
